# First evidence of mutualism between ancient plant lineages (Haplomitriopsida liverworts) and Mucoromycotina fungi and its response to simulated Palaeozoic changes in atmospheric CO_2_

**DOI:** 10.1111/nph.13024

**Published:** 2014-09-17

**Authors:** Katie J Field, William R Rimington, Martin I Bidartondo, Kate E Allinson, David J Beerling, Duncan D Cameron, Jeffrey G Duckett, Jonathan R Leake, Silvia Pressel

**Affiliations:** 1 Department of Animal and Plant Sciences, Western Bank, University of SheffieldSheffield, S10 2TN, UK; 2 Department of Life Sciences, Imperial College LondonLondon, SW7 2AZ, UK; 3 Jodrell Laboratory, Royal Botanic GardensKew, TW9 3DS, UK; 4 Department of Life Sciences, Natural History MuseumCromwell Road, London, SW7 5BD, UK

**Keywords:** carbon dioxide, *Endogone*, Haplomitriopsida, *Haplomitrium gibbsiae*, liverwort, Mucoromycotina, mycorrhiza, *Treubia lacunosa*

## Abstract

- The discovery that Mucoromycotina, an ancient and partially saprotrophic fungal lineage, associates with the basal liverwort lineage Haplomitriopsida casts doubt on the widely held view that Glomeromycota formed the sole ancestral plant–fungus symbiosis. Whether this association is mutualistic, and how its functioning was affected by the fall in atmospheric CO_2_ concentration that followed plant terrestrialization in the Palaeozoic, remains unknown.
- We measured carbon-for-nutrient exchanges between Haplomitriopsida liverworts and Mucoromycotina fungi under simulated mid-Palaeozoic (1500 ppm) and near-contemporary (440 ppm) CO_2_ concentrations using isotope tracers, and analysed cytological differences in plant–fungal interactions. Concomitantly, we cultured both partners axenically, resynthesized the associations *in vitro*, and characterized their cytology.
- We demonstrate that liverwort–Mucoromycotina symbiosis is mutualistic and mycorrhiza-like, but differs from liverwort–Glomeromycota symbiosis in maintaining functional efficiency of carbon-for-nutrient exchange between partners across CO_2_ concentrations. Inoculation of axenic plants with Mucoromycotina caused major cytological changes affecting the anatomy of plant tissues, similar to that observed in wild-collected plants colonized by Mucoromycotina fungi.
- By demonstrating reciprocal exchange of carbon for nutrients between partners, our results provide support for Mucoromycotina establishing the earliest mutualistic symbiosis with land plants. As symbiotic functional efficiency was not compromised by reduced CO_2_, we suggest that other factors led to the modern predominance of the Glomeromycota symbiosis.

## Introduction

The establishment of fungal symbioses has been widely considered one of the key innovations that facilitated plant terrestrialization 460–480 million yr ago (Ma), since this was first hypothesised by Pirozynski & Malloch (1975). Until recently, the ancestral plant–fungus symbiosis was assumed to involve members of the Glomeromycota based on four lines of evidence. First, Glomeromycota fungi were the only symbionts known to occur within basal land plant clades (the liverwort, hornwort and pteridophyte grades) on the basis of their characteristic intracellular arbuscules, vesicles and coils and largely aseptate hyphae (Ligrone, 1988; Selosse & Le Tacon, 1998; Read *et al*., 2000; Ligrone *et al*., 2007; Smith & Read, 2008). In contrast, ascomycete and basidiomycete symbioses are restricted to derived groups of liverworts and seed plants (Pressel *et al*., 2008, 2010; Smith & Read, 2008; Bidartondo & Duckett, 2010). Secondly, arbuscule-like structures and vesicles characterize fungi fossilized within early Devonian vascular land plants (Stubblefield *et al*., 1987; Remy *et al*., 1994; Taylor *et al*., 1995), lending weight to the hypothesis that Glomeromycota fungi played a pivotal role in the evolution of land plants (Pirozynski & Malloch, 1975; Malloch *et al*., 1980; Selosse & Le Tacon, 1998). Thirdly, molecular fungal phylogenies place Glomeromycota as an ancient monophyletic group sister to Ascomycota and Basidiomycota (James *et al*., 2006) or sister to Mucoromycotina (Lee & Young, 2009; Lin *et al*., 2014). Early molecular clock estimates suggested that the Glomeromycota diverged from other fungi 353–462 Ma (Simon *et al*., 1993), with more recent estimates pushing back to 460–600 Ma (Redecker *et al*., 2000; Redecker & Raab, 2006). Finally, bryophytes are the most basal extant land plants (although the exact order of divergence within the bryophytes remains under debate; see Cox *et al*., 2014) and fungi associating with some extant thalloid liverworts have been identified as members of the Glomeromycota (Ligrone *et al*., 2007; Humphreys *et al*., 2010; Field *et al*., 2012).

**Figure 1:**
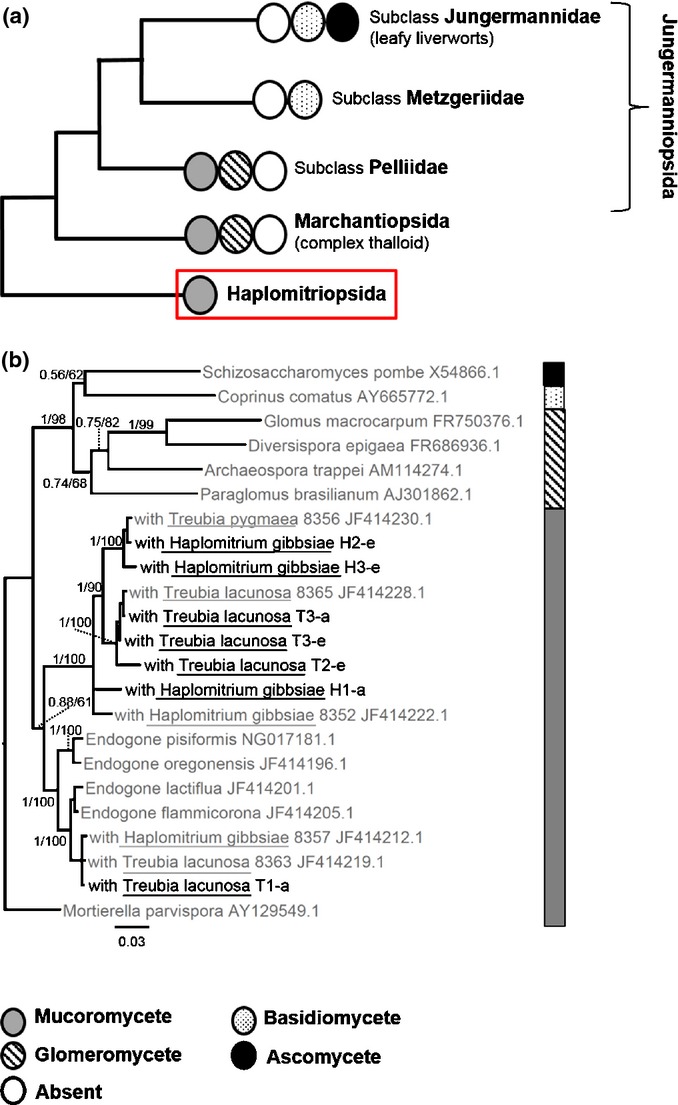
(a) Liverwort phylogeny showing key nodes alongside commonly associated fungal symbionts (James *et al*., 2006; Pressel *et al*., 2008, 2010; Bidartondo & Duckett, 2010; Humphreys *et al*., 2010; Bidartondo *et al*., 2011; Field *et al*., 2012; Desiró *et al*., 2013). The red box shows the clade investigated in the present study. (b) Bayesian inference to show the placement of experimental plant-symbiotic fungi within the fungal phylogeny. Black denotes experimental sequences while GenBank reference sequences are in grey. Values indicate support values from Bayesian inference/maximum likelihood. Sequence data are in Supporting Information Table S2.

We now know from additional molecular data derived using methods that enable detection of Mucoromycotina in addition to Glomeromycota, that the earliest diverging group of liverworts, the Haplomitriopsida including the genera *Treubia* and *Haplomitrium* (Heinrichs *et al*., 2005, 2007; Crandall-Stotler *et al*., 2009) (Fig. 1a,b), associate with Mucoromycotina (Bidartondo *et al*., 2011). The earliest divergent lineage of the Mucoromycotina is the order Endogonales, of poorly characterized ecology so far, with later genera including the saprotrophic *Umbellopsis*, *Rhizopus* and *Phycomyces*. Previously undetected Mucoromycotina fungi are now being observed within an increasing number of simple and complex thalloid liverworts (W. R. Rimington, unpublished) and are widespread in hornworts (Desiró *et al*., 2013), often occurring simultaneously with Glomeromycota fungi. These findings, together with the predominantly Gondwanan distribution of the plant–Mucoromycotina symbioses (Pressel *et al*., 2010), support the hypothesis that it was these, either instead of or simultaneously with plant–Glomeromycota symbioses, that were involved in the initial colonization of Earth's land-masses by plants (Bidartondo *et al*., 2011). Further testing of this hypothesis now requires elucidation of the functioning of plant–Mucoromycotina fungi associations and their anatomical characterization.

Physiological studies on the Glomeromycota-associated complex thalloid liverworts *Marchantia paleacea* and *Preissia quadrata* indicated that their fungal associations function in a similar mutualistic manner to those found in modern-day ‘higher’ plants (Humphreys *et al*., 2010; Field *et al*., 2012). Changes in the functional benefits of the symbiosis were investigated in these studies in the context of the high atmospheric CO_2_ concentration ([CO_2_]_a_) under which land plants originated, and the subsequent 90% [CO_2_]_a_ drop coincident with the diversification of terrestrial ecosystems through the Palaeozoic (Berner, 2006). In Field *et al*. (2012), the ratio of fungal-acquired phosphorus exchanged for plant photosynthate, termed ‘mycorrhizal efficiency’, was significantly lower under ambient compared with elevated [CO_2_]_a_.

These recent advances raise fundamental questions concerning the functioning of the Mucoromycotina fungal associates of Haplomitriopsida plants in comparison to Glomeromycota fungi and their responses to the changes in [CO_2_]_a_ that occurred as land plants evolved. In particular, the following questions are raised.

(1) Is the association of Mucoromycotina fungi with Haplomitriopsida liverworts a mutualistic symbiosis, with reciprocal exchange of fungal-acquired mineral nutrients for photosynthate like that found between Glomeromycota fungi and thalloid liverworts?
(2) If the Mucoromycotina–liverwort association is mutualistic, what is its efficiency relative to Glomeromycota–liverwort associations in terms of nutrient gain per unit of photosynthate invested in the fungal partners? A lower efficiency might explain why derived thalloid liverworts and most vascular plants associate with Glomeromycota.
(3) Does atmospheric CO_2_ differentially affect the functional efficiency of Mucoromycotina and Glomeromycota fungal associations with plants? Possible differences may provide some understanding as to why Mucoromycotina were largely replaced by Glomeromycota in later-diverging plant lineages.

**Figure 2:**
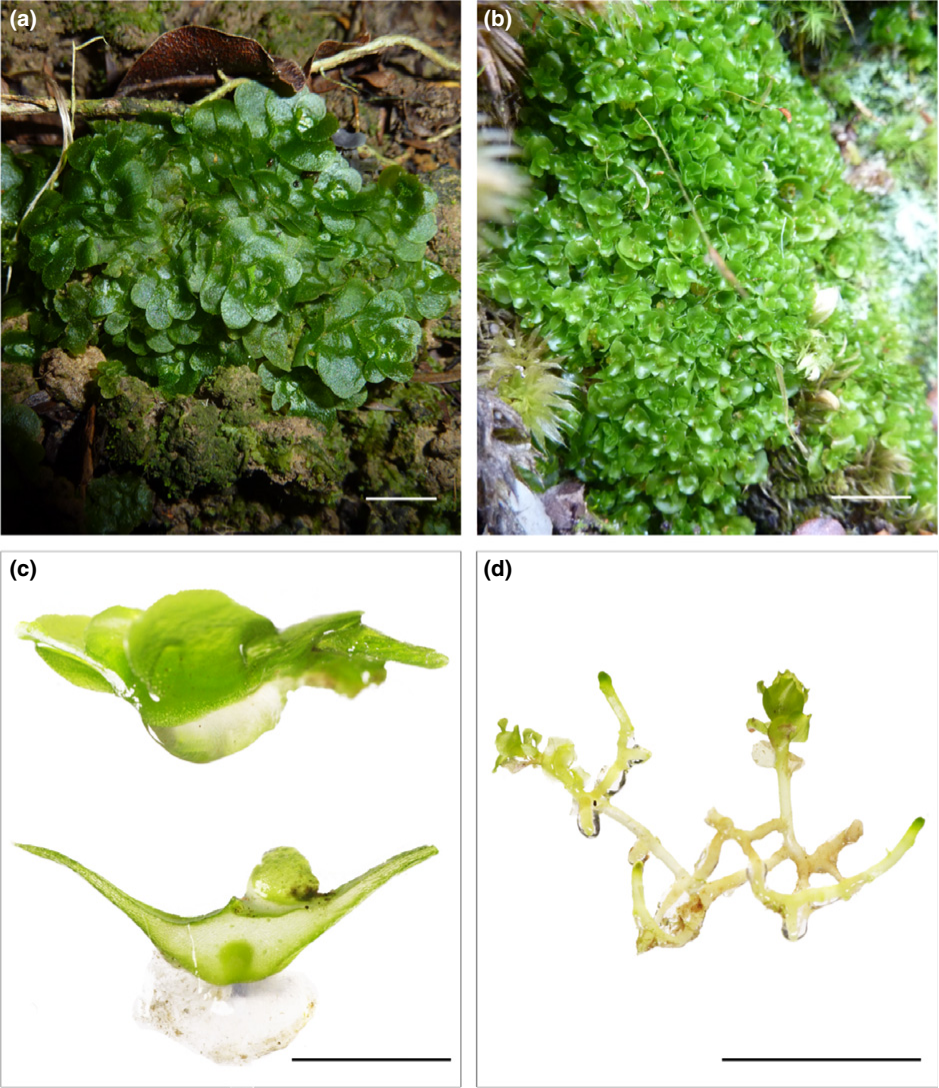
Liverworts of the Haplomitriopsida studied in the present investigation. (a, b) Whole plants of (a) *Treubia lacunosa* and (b) *Haplomitrium gibbsiae* photographed in the field. (c) Cleaned cross-section of *T. lacunosa* (bottom) and whole plant showing thick mucilage layer below fungal-colonized midrib. (d) Cleaned *H. gibbsiae* showing underground axes enveloped by copious mucilage. Bars 10 mm (in all panels).

To address these questions, we examined in detail the physiology and cytology of the fungal associations in the extant liverworts *Treubia lacunosa* (Fig. 2a,c) and *Haplomitrium gibbsiae* (Fig. 2b,d) under 440 versus 1500 ppm [CO_2_]_a_. We compared their functioning to that of the thalloid liverworts *P. quadrata* and *M. paleacea* in symbiosis with Glomeromycota fungi in a previous investigation carried out under identical experimental conditions (Field *et al*., 2012).

## Materials and Methods

### Plant species and growth conditions

We collected experimental stock plants of *Haplomitrium gibbsiae* (Steph.) and *Treubia lacunosa* (Col.) from the South Island of New Zealand in April 2012 (Fig. 2). Vouchers are deposited in the Natural History Museum, London, UK. Upon return to the UK, we transplanted collected plants into experimental pots, 120 mm diameter × 100 mm depth, containing acid-washed silica sand and 5% pot volume Irish *Sphagnum* moss-peat to aid water retention properties of the substrate and to provide minimal nutrients. Plants were watered with an artificial rainwater solution (see Supporting Information Table S1) to provide realistic levels of nutrient availability (Olsson & Tyler, 1993). Into each pot, we inserted three windowed, mesh-covered cores measuring 85 mm in length and 15 mm external diameter (Fig. S1). Based on the methods of Johnson *et al*. (2001), each core had two windows (20 mm × 50 mm) cut into the portion to be set into the substrate. The windows and base were covered by 10-μm pore size nylon mesh and sealed with a fast-setting cement (Polypipe Building Products, Doncaster, UK). This mesh size is fine enough to exclude liverwort rhizoids (20 μm), yet allows the ingrowth of fungal hyphae (< 5 μm). We filled two cores with a homogeneous mixture of acid-washed silica sand (89% core volume), native soil gathered from around the rhizoids and underground axes of both *H. gibbsiae* and *T. lacunosa* (10% core volume) and finely ground tertiary basalt rock grains (1% core volume) to act as fungal bait (Field *et al*., 2012). A perforated fine-bore Portex capillary tube (100 mm in length and 1.02 mm internal diameter; Fisher Scientific, Loughborough, UK) was installed in each of the soil-filled cores running the core's length. This was sealed using fast-setting cement 5 mm from the bottom of the core to prevent isotope leaching. The third core was filled with glass wool to enable belowground respiration sampling throughout the ^14^C-labelling period.

Plants were maintained under controlled environment conditions mimicking the natural light conditions experienced in the wild of 50 μmol m^−2^ s^−1^ (representing half-light-saturating conditions for nonvascular plants; Nobel, 1999; Fletcher *et al*., 2006), 70% relative humidity, 15°C : 12°C day : night temperatures and a 12-h day length. Experimental plants were grown either at 440 ppm [CO_2_]_a_ (*n *=* *10) or at 1500 ppm [CO_2_]_a_ (*n = *10). The [CO_2_]_a_ was monitored using CARBOCAP GMP343 CO_2_ sensors (Vaisala, Birmingham, UK) and maintained through addition of gaseous CO_2_. Pots were regularly rotated within cabinets. Cabinets and contents were alternated every 2 wk to avoid pseudo-replication. Plants were acclimated to chamber/growth regimes for 12 wk to allow establishment of mycelial networks (see Johnson *et al*., 2001).

### Molecular identification of fungal symbionts

We dissected living *T. lacunosa* and *H. gibbsiae* plants from the wild to isolate tissue areas (2–3 mm) where fungal colonization was highest; for *T. lacunosa* the central midrib was used, while in *H. gibbsiae* fungal-colonized underground axes were selected. Genomic DNA was extracted using the method of Gardes & Bruns (1993) in combination with the QBioGene Gene-Clean kit (Fisher Scientific). The universal fungal 18S primer combination NS1 and EF3 (White *et al*., 1990; Smit *et al*., 1999) was used with Sigma JumpStart and the following PCR settings: 94°C for 2 min, 34 cycles of 94°C for 30 s, 53°C for 30 s and 72°C for 1 min 30 s, followed by a 72°C final step for 7 min. These primers amplify all known lineages of Mucoromycotina and Glomeromycota (Bidartondo *et al*., 2011; Desiró *et al*., 2013). The PCR products were cloned using the Invitrogen TOPO TA cloning kit (Life Technologies, Paisley, UK) and at least eight colonies from two independent DNA extractions per plant were used for sequencing. Affymetrix ExoSAP-IT (Santa Clara, CA, USA) was used to purify PCR products and DNA was sequenced using Big Dye v3.1 on an Applied Biosystems genetic analyser (ABI3730) with NS1, following purification by ethanol and EDTA precipitation. Sequences were initially identified using NCBI BLAST (National Centre for Biotechnology; Altschul *et al*., 1997) and selected for further analysis based on their most closely related sequences. All the sequences from one sample predicted to be Mucoromycotina by BLAST were aligned. Sequences found to be significantly different from one another were chosen for further sequencing using NS3 and NS5 (White *et al*., 1990) and further analysis. Editing and assembly into contigs of *c*. 1700 bp were performed in geneious v5.6.4 (Biomatters, Auckland, New Zealand) and muscle alignment algorithms (Edgar, 2004) were used within mega v. 5.1 (Tamura *et al*., 2011). Reference DNA sequences were obtained from GenBank (Benson *et al*., 2005). uchime (Edgar *et al*., 2011) was used within mothur (Schloss *et al*., 2009) to test for chimeric sequences. Maximum likelihood phylogenies were produced with mega using 1000 bootstrap replicates and 95% site coverage cut-off. Evolutionary models were tested in mega. The model with the lowest Bayesian information criterion value was selected (TN93 + G). Bayesian inference was carried out using MrBayes (Huelsenbeck & Ronquist, 2001), with a HKY85 model (nst = 2) and gamma rates as the evolutionary model. The consensus tree produced by MrBayes was visualized and edited using FigTree v1.4 (Rambaut & Drummond, 2012).

### Quantification of fluxes of C, ^33^P and ^15^N between liverworts and fungi

Following the 12-wk plant acclimation period, we introduced 100 μl of ^33^P-labelled aqueous solution of orthophosphate (0.5 MBq) and ^15^N-ammonium chloride (1 mg ml^−1^) into one of the soil-filled mesh cores in each pot via the installed capillary tubing to ensure even distribution of the isotope in each core (see Fig. S1a). Control cores in each pot were injected with 100 μl of distilled H_2_O. In half of the experimental pots, cores in which isotope labels were introduced were left static to preserve direct hyphal connections with the liverworts. In the remaining half, labelled cores were rotated through 90° daily to sever the hyphal connections between the core and plants. By subtracting the quantity of isotope present in plants in pots where hyphal connections had been severed, we were able to account for movement of isotopes into and out of soil cores through diffusion.

After 21 d, the soil cores were sealed with anhydrous lanolin and plastic caps (see Fig. S1b). The cores containing glass wool were sealed with a rubber septum (SubaSeal; Sigma) to allow below-ground respiration gas sampling throughout the labelling period. Each pot was then sealed into a gas-tight labelling chamber and a 1.1 MBq ^14^CO_2_ pulse liberated via addition of 2 ml of 10% lactic acid to 15 μl of Na^14^CO_3_ (2 GBq mmol^−1^). Pots were maintained under growth chamber conditions and 1 ml of labelling chamber headspace gas was sampled after 1 h and every 4 h thereafter. Gas was sampled from below ground via the glass-wool filled core after 1 h and every 2 h thereafter to monitor below-ground respiration and ^14^C flux through the plant–mycelial network. Gas samples were injected directly into gas-evacuated scintillation vials containing 10 ml of Carbosorb (Perkin Elmer, Beaconsfield, UK) to trap CO_2_ within the sample. Ten millilitres of Permafluor (Perkin Elmer) was added to these and radioactivity within the sample was measured directly through liquid scintillation counting (Packard Tri-carb 3100TR; Isotech, Chesterfield, UK).

Pots were incubated under cabinet conditions (see ‘Plant species and growth conditions’) until maximal ^14^C flux was detected in below-ground gas samples (17 h). At this point all cores were removed from pots and incubated in gas-tight containers with 2 ml of 2 M KOH to trap ^14^CO_2_ emitted through respiration. Cores were transferred to fresh containers containing KOH every 2 h for a further 6 h. One millilitre of the KOH that absorbed CO_2_ was transferred to a scintillation vial containing 10 ml of Ultima Gold liquid scintillant (Perkin Elmer) and radioactivity was measured using liquid scintillation counting (Packard Tri-carb 3100TR; Isotech).

### Plant harvest and tissue analyses

After removal of soil cores from pots, plant and soil materials were separated, freeze-dried and weighed. Plant tissues were homogenized and subsamples weighed out to between 10 and 30 mg (correct to 0.001 mg) and digested in 1 ml of concentrated sulphuric acid at 365°C for 15 min. When cool, 100 μl of hydrogen peroxide was added to each sample and reheated to 365°C until clear. We then diluted the digest solution to 10 ml with distilled water. Two millilitres of the diluted digest was then added to 10 ml of Emulsify-safe (Perkin Elmer). The radioactivity of the sample was measured directly using liquid scintillation counting (Packard Tri-carb 3100TR; Isotech) and normalized to the specific mass of digested tissue. The ^33^P transferred from fungus to plant was determined using published equations (Cameron *et al*., 2007):

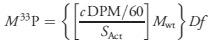
Eqn 1

(*M*^33^P, mass of ^33^P (mg); *c*_DPM,_ counts as disintegrations per minute; *S*_Act_, specific activity of the source (Bq mmol^−1^); *Df*, dilution factor; *M*_wt_, molecular mass of P.)

The ^15^N transfer from fungus to plant was detected and quantified using continuous-flow mass spectrometry (2020 Isotope Ratio Mass Spectrometer (PDZ Europa, Northwich, UK) coupled to a PDZ ANCA GSL preparation unit). Data were collected as atom % ^15^N and as % N using unlabelled control plants for background detection. The plant tissue concentration of ^15^N was calculated using the methods of Cameron *et al*. (2006).

Total carbon (^12^C + ^14^C) fixed by the plant and transferred to the fungal network was calculated as a function of the total volume and CO_2_ content of the labelling chamber and the proportion of the supplied ^14^CO_2_ label fixed by the plants. The difference in carbon between the static and rotated cores is equivalent to the total carbon transferred from the plant to the symbiotic fungus within the soil core. Total carbon assimilated by the plant was calculated using Eqn 2 (Cameron *et al*., 2006):

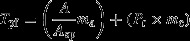
Eqn 2

where *T*_pf_ is the transfer of carbon from plant to fungus, *A* is the radioactivity of the tissue sample (Bq), *A*_sp_ is the specific activity of the source (Bq Mol^−1^), *m*_a_ is the atomic mass of ^14^C, *P*_r_ is the proportion of the total ^14^C label supplied present in the tissue, and *m*_c_ is the mass of carbon in the CO_2_ present in the labelling chamber (g) (from the ideal gas law; Eqn 3):

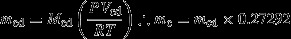
Eqn 3
(*m*_cd_, mass of CO_2_ (g); *M*_cd_, molecular mass of CO_2_ (44.01 g mol^−1^); *P*, total pressure (kPa); *V*_cd_, volume of CO_2_ in the chamber (0.003 m^3^); *R*, universal gas constant (J K^−1^ mol^−1^); *T*, absolute temperature (K); *m*_c_, mass of carbon in the CO_2_ present in the labelling chamber (g), where 0.27292 is the proportion of carbon in CO_2_ on a mass fraction basis (Cameron *et al*., 2008).)

### Ultrastructural analyses of plant–fungal associations

We processed wild-collected and experimental plants (grown at each [CO_2_]_a_) for transmission electron microscopy (TEM) and scanning electron microscopy (SEM). For TEM, thalli were fixed in 3% glutaraldehyde, 1% fresh formaldehyde and 0.75% tannic acid in 0.05 M Na-cacodylate buffer, pH 7, for 3 h at room temperature. After rinses in 0.1 M buffer, samples were post-fixed in buffered (0.1 M, pH 6.8) 1% osmium tetroxide overnight at 4°C, dehydrated in an ethanol series and embedded in low-viscosity resin via ethanol (TAAB Laboratories Equipment Ltd, Aldermarston, UK). Thin sections were cut with a diamond knife, stained with methanolic uranyl acetate for 15 min and in Reynolds’ lead citrate for 10 min, and observed with a Hitachi H-7100 transmission electron microscope at 100 kV (Hitachi High-Technologies Europe, Maidenhead, UK). For SEM, we fixed thalli in 3% glutaraldehyde; they were then dehydrated through an ethanol series, critical-point dried using CO_2_ as transfusion fluid, sputter coated with 390-nm palladium-gold and viewed using a FEI Quanta scanning electron microscope (FEI, Hillsboro, OR, USA).

For light microscopy, 0.5-μm-thick sections, cut with a diamond Histo Knife (DiaTOME, Biel, Switzerland), were stained with 0.5% toluidine blue and photographed with a Zeiss Axioskop light microscope fitted with an MRc Axiocam digital camera.

### Axenic culture of liverworts and fungal isolates and recolonization of *H. gibbsiae*

#### Axenic plants

*Haplomitrium gibbsiae* and *T. lacunosa* were cultured *in vitro* by modifying methods developed for isolating fungi from other bryophytes (Duckett *et al*., 2004). Undehisced mature capsules were removed from sporophytes, rinsed in water and then surface-sterilized in 1% sodium hypochlorite for 2 min. Sterilized capsules were broken open and the spores spread onto 1/100 Parker medium (Klekowski, 1969) solidified with 1% Phytagel (Sigma-Aldrich) where they germinated within 20 d. Cultures were maintained at 18°C, with a day : night regime of 14 h : 10 h, at an irradiance of 100 μmol m^−2^ s^−1^. Following germination, plants were regularly subcultured (every 3 months) onto fresh medium and have now been maintained for over 2 yr.

#### Isolation of fungal symbionts

*Treubia lacunosa* thalli from the wild were cleaned of adhering substratum and the rhizoids, marginal lobes, lobules and dorsal regions removed, leaving only the fungus-colonized midrib region. After rinsing in running water for 3 min, excised midrib regions were surface-sterilized for 1 min in 1% sodium hypochlorite. Sterile midrib pieces were cut into *c*. 0.5-mm sections, placed onto a novel fungal medium (described in the next paragraph) and incubated in the dark at 20–22°C. For isolation of the fungus in *H. gibbsiae*, subterranean axes were surface-sterilized as detailed above for *T. lacunosa*.

The fungal medium consisted of glucose, 10 g; KH_2_PO_4_, 0.5 g; NH_4_Cl, 0.25 g; MgSO_4_.7H_2_O, 0.15 g; 1% ferric citrate solution, 0.5 ml; CaCl_2_.2H_2_O, 0.05 g; NaCl, 0.025 g; chloramphenicol, 50 mg; thiamin HCl, 100 mg; biotin, 25 μg; n-inositol, 10 μg; agar, 15 g; in 1 l of double-deionized water. This was developed as a modification of the medium used previously for isolation from *Endogone* fruit bodies (Berch & Fortin, 1983) and contains a high concentration of thiamin (Dalpe, 1990). After autoclaving and cooling to 45°C, 50 mg of streptomycin, 50 mg of ampicillin and 1 mg of benomyl (dissolved in DMSO) were added using a sterile 0.2-μm filter. Once fungal outgrowths from the plant fragments became visible (within 2–3 wk), we subcultured hyphae onto fungal medium without antibiotics to maximize growth.

#### Recolonization of asymbiotic plants by isolated fungi

We placed plugs of fungal hyphae growing on fungal medium adjacent to axenic *H. gibbsiae* gametophytes on 1/100 Parker medium (Klekowski, 1969). The host and fungi were grown together under the conditions described previously for plants. Plates were undisturbed for 3 months to allow time for the slow-growing fungus to colonize the plants. Tissue was harvested and prepared for SEM (see ‘Ultrastructural analyses of plant-fungal associations’).

### Statistics

Effects of plant species, [CO_2_]_a_ and the interaction between these factors on the C, ^33^P and ^15^N fluxes between plants and fungi were tested using ANOVA with additional *post hoc* Tukey testing where indicated. All statistical analyses were carried out using minitab version 12.21 (Minitab Inc., PA, USA).

## Results

### Molecular identification of fungi

Molecular identification of fungal partners (from both the wild-collected plants and the treated plants at the end of the experiments) confirmed that both *H. gibbsiae* and *T. lacunosa* were colonized by Mucoromycotina fungi (Fig. 1b). The fungi identified here were the same as found previously in wild populations of the same species (Bidartondo *et al*., 2011). Similarly, axenic fungal isolates from *T. lacunosa* have DNA sequences identical to those from Mucoromycotina fungi identified within wild-collected plants. Sequences are deposited in GenBank (accession numbers: KJ921770–KJ921776).

### Reciprocal carbon-for-nutrient transfer and assimilation

In order to determine the symbiotic status of liverwort–Mucoromycotina fungi associations, we investigated whether there was reciprocal exchange of fungal-acquired mineral nutrients for photosynthate.

#### Liverwort-to-fungus carbon transfer

**Figure 3:**
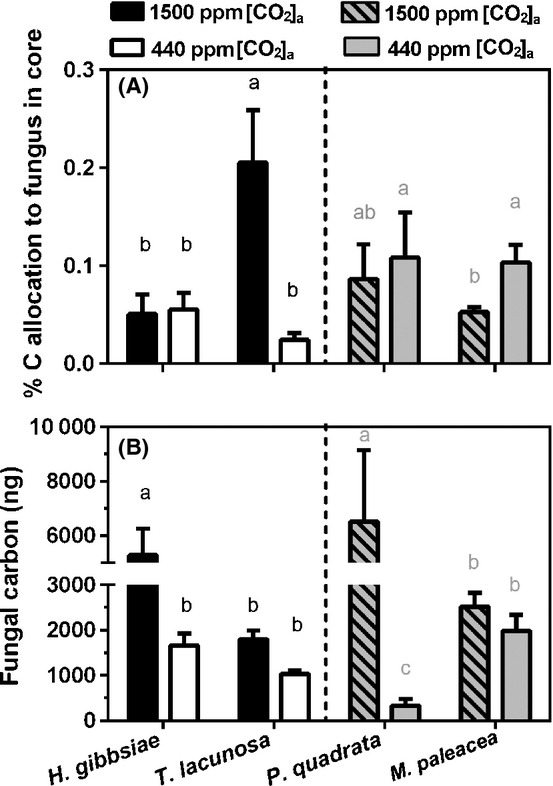
(A) Percentage allocation of plant-fixed carbon to mycorrhizal hyphal network in mesh-walled cores and (B) total measured plant-derived carbon allocated to the mycorrhizal hyphal network in soil cores under both simulated Palaeozoic (1500 ppm) [CO_2_]_a_ (black bars) and near-contemporary ambient (440 ppm) [CO_2_]_a_ (white bars). Grey shaded bars represent previously published data for thalloid liverwort species (Field *et al*., 2012). Error bars, + SE. Different letters denote statistical significance where *P *< 0.05 (Tukey's post hoc test). *H. gibbsiae*, *Haplomitrium gibbsiae*; *T. lacunosa*, *Treubia lacunosa*; *P. quadrata*, *Preissia quadrata*; *M. paleacea*, *Marchantia paleacea*.

By tracing and quantifying the flow of ^14^C from plant to fungus in liverwort specimens transplanted from the field, we calculated both the percentage of plant-fixed carbon allocated to the Mucoromycotina fungal mycelia in the soil (Fig. 3A) and the total amount of carbon received by these fungal networks (Fig. 3B). These results are displayed alongside published data (Field *et al*., 2012) for *M. paleacea* and *P. quadrata* in symbiosis with fungi of the Glomeromycota that were grown under identical conditions and growth chambers as in the present study.

*Treubia lacunosa* allocated a greater percentage of photosynthate to Mucoromycotina fungi under 1500 ppm [CO_2_]_a_ compared with 440 ppm [CO_2_]_a_. There was no difference in percentage photosynthate carbon allocation between [CO_2_]_a_ treatments for *H. gibbsiae* (Fig. 3A). In terms of absolute amount of carbon transferred to mycelial networks in mesh cores, both Haplomitriopsida liverworts allocated more carbon under elevated [CO_2_]_a_, a pattern of response similar to that of *Glomus* partners in *P. quadrata* and *M. paleacea* (Fig. 3B) (Field *et al*., 2012).

#### Fungal transfer of ^33^P and ^15^N to host liverworts

**Figure 4:**
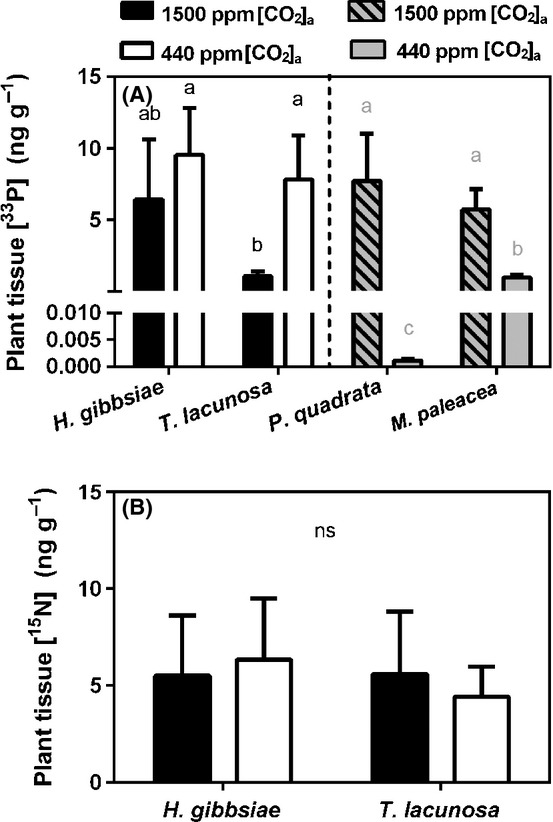
(A) Plant tissue concentration of ^33^P assimilated via fungal symbionts at 1500 ppm [CO_2_]_a_ (black bars) and 440 ppm [CO_2_]_a_ (white bars). (B) Concentration of fungal-assimilated ^15^N in plant tissues grown at 1500 ppm [CO_2_]_a_ (black bars) and 440 ppm [CO_2_]_a_ (white bars). Grey shaded bars represent previously published data for thalloid liverwort species with Glomeromycota mycorrhiza-like associations (Field *et al*., 2012). Error bars, + SE. Different letters on bars represent *P *<* *0.05 (Tukey‘s post hoc test). ns, not significant. *H. gibbsiae*, *Haplomitrium gibbsiae*; *T. lacunosa*, *Treubia lacunosa*; *P. quadrata*, *Preissia quadrata*; *M. paleacea*, *Marchantia paleacea*.

In contrast to previous findings in liverworts with Glomeromycota partners, the transfer of nutrients to plants from rhizoid-excluding soil cores via Mucoromycotina fungi did not appear to be positively affected by higher [CO_2_]_a_ (Fig. 4A,B). All plants gained ^33^P under both elevated and ambient [CO_2_]_a_ (Fig 4A), *T. lacunosa* gaining least when grown at 1500 ppm [CO_2_]_a_.

The two Haplomitriopsida liverworts received ^15^N from their fungal partners under both [CO_2_]_a_ treatments and there was little difference between species in terms of tissue ^15^N concentration or [CO_2_]_a_ treatment effects (Fig. 4B, Table 1).

**Table 1:** Summary of differences in mycorrhizal functionality (*F* ratio from ANOVA) in *Haplomitrium gibbsiae* and *Treubia lacunosa* at elevated [CO_2_]_a_ (1500 ppm) and ambient [CO_2_]_a_ (440 ppm)

	Plant species	CO_2_ treatment	Species × CO_2_
Total fungal carbon (ng)	10.57**	6.92*	4.34*
% plant-fixed carbon allocated to fungus	15.31***	4.31*	6.85*
[^33^P] in plant tissue (ng g^−1^)	2.56	1.33	0.35
[^15^N] in plant tissue (ng g^−1^)	0.22	0.54	0.00
P-for-C efficiency (ng ng^−1^)	0.51	0.28	0.34
N-for-C efficiency (ng ng^−1^)	0.33	2.68	0.01

All ANOVA have 1, 23 df for total carbon and % carbon allocation, and 1, 15 df for nutrient and efficiency measures.

* , *P *<* *0.05;

** , *P *<* *0.01;

*** , *P *<* *0.001; post hoc Tukey test (*n *=* *10 and 5).

#### Carbon-for-nutrient exchange efficiency

We calculated the carbon-for-nutrient exchange efficiency of the liverworts using the measured total carbon fixed and transferred to fungal partners in the mesh-walled soil cores and the absolute ^33^P or ^15^N content of plant material within each pot.

**Figure 5:**
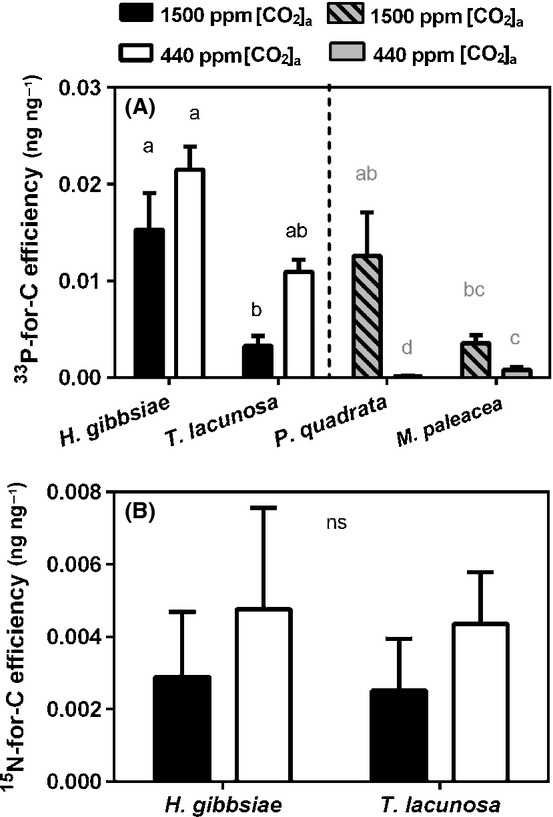
(A) ^33^Phosphorus-for-carbon efficiency. Grey shaded bars represent previously published data for thalloid liverwort species with Glomeromycota mycorrhiza-like associations (Field *et al*., 2012). (B) ^15^N-for-carbon efficiency for each liverwort species at both 1500 ppm [CO_2_]_a_ (black bars) and 440 ppm [CO_2_]_a_ (white bars). Error bars, + SE. Different letters indicate *P *<* *0.05 (Tukey‘s post hoc test). ns, not significant. *H. gibbsiae*, *Haplomitrium gibbsiae*; *T. lacunosa*, *Treubia lacunosa*; *P. quadrata*, *Preissia quadrata*; *M. paleacea*, *Marchantia paleacea*.

In Haplomitriopsida liverworts, there was no significant effect of [CO_2_]_a_ on carbon-for-phosphorus exchange efficiency. However, there was a trend for reduced efficiency in each of the species tested at 1500 ppm [CO_2_]_a_, opposite to that observed in previous experiments using thalloid liverworts in symbiosis with Glomeromycota fungi (Fig. 5A). We observed no differences in carbon-for-nitrogen exchange efficiency between the two Haplomitriopsida liverworts with respect to growth in [CO_2_]_a_ treatments, but there was a trend for greater efficiency at the lower [CO_2_]_a_ (Fig. 5B).

### Cytology of fungal association

#### Wild-collected plants, and plants grown at 440 and 1500 ppm [CO_2_]_a_

Overall, the cytology of fungal colonization in plants grown at 440 and 1500 ppm [CO_2_]_a_ was the same as that of their wild counterparts characterized previously in both *T. lacunosa* (Duckett *et al*., 2006) and *H. gibbsiae* (Carafa *et al*., 2003), but with only modest differences in relation to [CO_2_]_a_ treatments.

#### Treubia lacunosa

**Figure 6:**
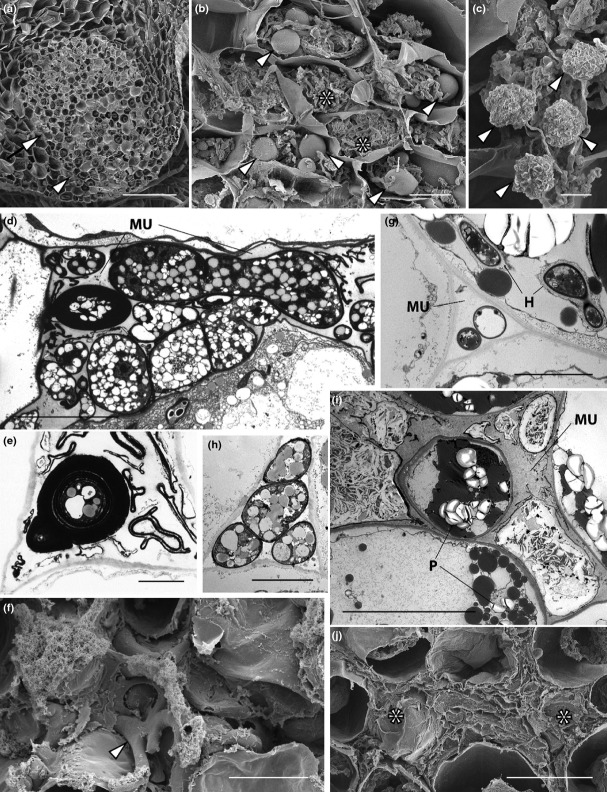
*Treubia lacunosa* grown at 440 and 1500 ppm [CO_2_]_a_. Scanning (a–c, f, j) and transmission (d, e, g–i) electron micrographs are shown. There was no change in the overall distribution of fungal colonization and in the cytology of the intracellular fungus between [CO_2_]_a_ treatments, both illustrated here in plants grown at 1500 ppm [CO_2_]_a_ (a–c). (a) Intracellular (arrowhead) and intercellular (arrow) fungal zones in the thallus midrib. (b) Thallus cells packed with hyphal coils (*) and young fungal lumps (arrowed). (c) Highly shrunken lumps (arrows). In plants grown at 440 ppm [CO_2_]_a_ (d–f) the mucilage-filled (MU) intercellular spaces are packed with fungus forming semi-parenchymatous structures (d, arrowed in f) as well as structures with thick, multilayered cell walls (e). In plants grown at 1500 ppm [CO_2_]_a_ (g–j) healthy intercellular hyphae are thin-walled (g, h) and present exclusively in the mucilage-filled (MU) small intercellular spaces adjacent to intracellular hyphae (H, in g). All other hyphae in the mucilage-filled intercellular zone (MU) are degenerate. Note the healthy host cells with intact plastids (P) (i). (j) SEM confirms the highly disrupted nature of the intercellular hyphae (*). Bars: (a) 200 μm; (b, f, i, j) 20 μm; (c, d, g, h) 5 μm; (e) 2 μm.

Fungal colonization exhibited the same overall distribution in plants grown at 440 and 1500 ppm [CO_2_]_a_ (Fig. 6a–c). In both [CO_2_]_a_ treatments (but illustrated here only in the 1500-ppm plants), fungal colonization occurred in a well-defined fungal zone in the thallus midrib (Fig. 6a), with the lowermost ventral layers, which lacked intercellular spaces, harbouring intracellular fungal lumps (or swellings) at various stages of development, and hyphal coils (Fig. 6b,c). Above this intracellular fungal zone lay a strictly intercellular one and it was in this zone that differences were observed between [CO_2_]_a_ treatments. In plants grown at 440 ppm [CO_2_]_a_ (Fig. 6d–f), living hyphae occupied the mucilage-filled spaces characteristic of this zone and formed large pseudo-parenchymatous structures (Fig. 6d), as well as structures with thick multi-layered walls (Fig. 6e). However, in plants grown at 1500 ppm [CO_2_]_a_ (Fig. 6g–j) healthy intercellular hyphae were invariably thin-walled and were restricted to the small intercellular spaces immediately adjacent to the intracellular fungus (Fig. 6g,h). All other intercellular hyphae were highly disrupted (Fig. 6i). Their degraded cytoplasm contained numerous crystalline profiles. Thick-walled intercellular structures like those observed in the intercellular spaces of plants grown at 440 ppm [CO_2_]_a_ (Fig. 6e) and in wild *T. lacunosa* (Duckett *et al*., 2006) were never observed. The same differences in the cytology of the intercellular fungus between [CO_2_]_a_ treatments obtained by TEM analyses were also observed under the SEM (Fig. 6f,j).

#### Haplomitrium gibbsiae

**Figure 7:**
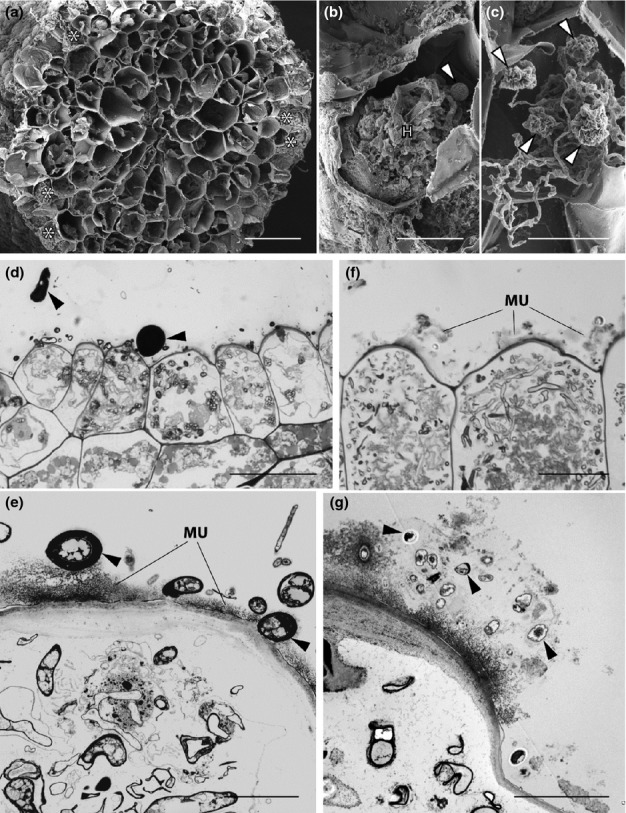
*Haplomitrium gibbsiae* grown at 440 and 1500 ppm [CO_2_]_a_. Light micrographs (d, f) and scanning (a–c) and transmission (e–g) electron micrographs are shown. Both the distribution and cytology of the association remained the same between [CO_2_]_a_ treatments and are illustrated here in plants grown at 1500 ppm [CO_2_]_a_ (a–c). (a) Transverse section of an underground axis; the fungus is restricted to the epidermal cells (*). (b) Intracellular hypha coil (H) and young lump (arrowed). (c) Highly shrunken lumps (arrowed). In plants grown at 440 ppm [CO_2_]_a_ (d, e) hyphae and thick-walled fungal structures (arrowed) are also present in the mucilage layer that envelops the underground axes. In plants grown at 1500 ppm [CO_2_]_a_ (f, g) only thin-walled fungal hyphae (arrowed) are present in the external mucilage (MU). Bars: (d) 500 μm; (f) 200 μm; (a) 100 μm; (b, c) 20 μm; (e, g) 5 μm.

Both the distribution and cytology of the association remained the same overall in plants grown at 440 and 1500 ppm [CO_2_]_a_ (Fig. 7a–c). In both [CO_2_]_a_ treatments (but illustrated here only in the 1500-ppm plants), fungal colonization was restricted to the epidermal layers of the underground axes and was strictly intracellular (Fig. 7a), with abundant hyphal coils and lumps at various stages of development filling the host epidermal cells (Fig. 7b,c). In plants grown at 440 ppm [CO_2_]_a_ (Fig. 7d,e), abundant fungal structures were also present in the copious mucilage that enveloped, and was produced by, the underground axes (Figs 2d, 7d,e). These consisted mainly of thick-walled fungal structures with a few thin-walled hyphae (Fig. 7d,e). A major difference between ambient and elevated [CO_2_]_a_-grown plants was that, in the latter, the hyphae in the extracellular mucilage layer were invariably thin-walled and we never observed the thick-walled structures that characterized wild plants and those grown at 440 ppm [CO_2_]_a_ (Fig. 7f,g).

### Axenic culture of liverworts and fungi and *in vitro* recolonization of *Haplomitrium gibbsiae* by Mucoromycotina fungi

**Figure 8:**
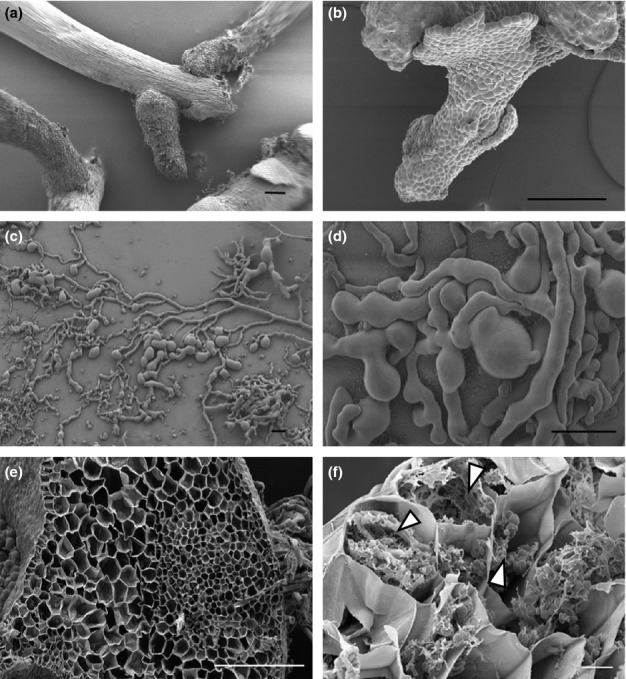
Scanning electron micrographs of axenically grown liverworts and fungal isolates and recolonization of *Haplomitrium gibbsiae* with Mucoromycotina fungi. (a) Wild-grown *H. gibbsiae* with Mucoromycotina fungi within underground axes. (b) *Haplomitrium gibbsiae* grown axenically showing failure to produce underground axes in the absence of a fungal partner in culture. (c, d) Mucoromycotina fungi in axenic culture showing characteristic lumps and thin hyphae. (e) Axenic *T. lacunosa*; cross-section of thallus completely lacking mucilage-filled intercellular spaces. (f) *In vitro* axenic culture with intracellular recolonization of *H. gibbsiae* epidermal cells by fungi of the Mucoromycotina (arrowed). Bars: (a) 200 μm; (b) 400 μm; (c, d) 20 μm; (e) 400 μm; (f) 10 μm. Sequence data are in Supporting Information Table S2; fungal isolate is identical to T1-a in Fig. 1(b).

*Haplomitrium gibbsiae* grown in axenic culture failed to produce the naked mucilage-invested underground axes characteristic of wild-collected plants (Fig. 8a,b). Instead, only green tissue identical to the above-ground axes observed in wild-collected plants was produced (Fig. 2b,d). The gross morphology of *T. lacunosa* in axenic culture was similar to that of wild-collected plants (Fig. 2a,c); however, asymbiotic plants lacked intercellular spaces (Fig. 8e) and produced little mucilage. Growth of fungi-free gametophytes was also slower than that of plants growing in symbiosis with Mucoromycotina fungi (i.e. in the experimental pots).

Axenic fungal isolates from *T. lacunosa* (Fig. 8c) comprised fine hyphal networks with terminal swellings and lump-like structures (Fig. 8d). No vesicles or coils were produced in culture.

Recolonization occurred (Fig. 8f) within 3 months of adding the fungal isolates to axenically grown *H. gibbsiae*. In recolonized plants, Mucoromycotina fungi invaded the apical regions of stems whose rudimentary leaves went on to grow into mucilage-invested naked axes, similar to those observed in wild-collected plants. Fungal recolonization was restricted to epidermal cells where it formed typical swellings/lumps and coils, characteristic of Mucoromycotina symbiotic associations in wild-collected plants (Fig. 8f).

## Discussion

The present study provides the first assessment of symbiotic functioning between the extant earliest diverging liverworts, Haplomitriopsida (Fig. 1a), and Mucoromycotina fungi (Fig. 1b). Isotope tracer studies showed that these associations were mutually beneficial, comparable to associations between complex thalloid liverworts and Glomeromycota symbionts reported previously (Humphreys *et al*., 2010; Field *et al*., 2012). The intracellular phases in both kinds of plant–fungal association were functionally analogous in facilitating reciprocal exchange of plant photosynthate for fungal-acquired nutrients from soil (Figs 4). These findings are further underpinned by our fulfilling of Koch's postulates for mutualistic symbiosis (Read *et al*., 2000) through establishing axenic cultures of both *H. gibbsiae* and isolated fungi, and re-establishing the symbiosis between the partners *in vitro*. Thus, the association between Haplomitriopsida liverworts and Mucoromycotina fungi is both mutualistic and mycorrhiza-like in plants lacking roots.

The morphological changes observed in axenic plants, that is the lack of mucilage secretion in both species, the lack of intercellular spaces in *Treubia*, and notably the lack of subterranean axes in *Haplomitrium*, reflect morphogenetic modifications by the fungal symbiont. As far as we are aware, these changes have no counterpart in Glomeromycota symbiosis with liverworts or vascular plants.

Our findings are timely following the recent report that a Devonian early vascular plant fossil, *Horneophyton ligneri,* dating to 411 Ma (Parry *et al*., 2011), harboured fungi with affinities to both Glomeromycota and Mucoromycotina (Strullu-Derrien *et al*., 2014). Diverse lines of evidence now place fungi of the Mucoromycotina as potential key players in the earliest symbiotic events between plants and fungi during the initial colonization of Earth's land-masses. Although the possibility cannot be excluded that symbiosis with Mucoromycotina fungi is a derived trait acquired through convergence or reversion, this appears unlikely given the presence of such fungi in Devonian fossils and our demonstration that associations between Mucoromycotina and extant basal land plants are mutualistic. Thus, our results strengthen the emerging view that fungi of the Mucoromycotina facilitated the evolution and diversification of early land plants, and may have been among the first fungi to form mutualisms with plants (Bidartondo *et al*., 2011). We add the caveat that, as the responses of extinct early land plant–fungal symbioses are unknown, we are restricted to assessing phenotypic plasticity of extant species (recognizing that these are a product of evolutionary adaptation) set within an evolutionary context.

There were striking differences in the way Mucoromycotina fungi–liverwort symbioses responded to simulating the major decline in [CO_2_]_a_ that occurred through the mid-late Palaeozoic, compared with previous studies of liverworts colonized by Glomeromycota fungi (Field *et al*., 2012). Our findings do not support our initial hypothesis that sharply declining [CO_2_]_a_ through the Palaeozoic would impair the efficiency of Mucoromycotina–plant symbiosis to a greater extent than that of Glomeromycota–plant symbiosis, favouring replacement by the latter in later diverging plants. While liverworts with Mucoromycotina fungal associations fixed more carbon and increased its allocation to symbiotic fungal networks under 1500 ppm compared with 440 ppm [CO_2_]_a_ (Fig. 3b), the plants did not receive proportionally more mineral nutrients in return (Fig. 4a,b). The efficiency of phosphorus-for-carbon exchange between Haplomitriopsida liverworts and Mucoromycotina compared with that between thalloid liverworts and Glomeromycota (Fig. 5a; Field *et al*., 2012) reveals much greater efficiency of the Mucoromycotina, especially under near-contemporary 440 ppm [CO_2_]_a_. This may explain why Haplomitriopsida liverworts are associated with Mucoromycotina fungi instead of Glomeromycota, but fails to explain why later diverging thalloid liverworts associate with Glomeromycota. The efficiencies of liverwort–Glomeromycota associations declined dramatically under 440 ppm [CO_2_]_a_ compared with the mid-Palaeozoic 1500 ppm [CO_2_]_a_, while the phosphorus-for-carbon and nitrogen-for-carbon exchange efficiencies of liverwort–Mucoromycotina associations remained the same or tended to increase at the lower [CO_2_]_a_ (Fig. 5a,b).

High [CO_2_]_a_ appeared to benefit the Mucoromycotina fungal partners, with increased percentage photosynthate allocation from *T. lacunosa* (Fig. 3a) and increased total carbon allocation from *H. gibbsiae* (Fig. 3b). This suggests that the simulated mid-Palaeozoic high [CO_2_]_a_ provided conditions that strongly selected for Mucoromycotina fungi to switch from saprotrophy to mutualistic associations with plants able to provide abundant and reliable supplies of photosynthate in return for assisting the uptake of plant growth-limiting nutrients. The facultative biotrophic capabilities of these fungi were confirmed by the slow growth of axenic cultures isolated from *T. lacunosa* tissue – such plant-independent growth has never been achieved for any Glomeromycota fungi beyond early stages of spore germination leading to root colonization. The extent to which plant-mutualistic Mucoromycotina fungi can grow without symbionts or even as saprotrophs in nature remains to be determined. Genomic investigations of Mucoromycotina may give clues as to whether symbiosis with plants has resulted in a loss of capacity to degrade and digest plant biopolymers as carbon sources, as this appears to underpin the obligately biotrophic nature of Glomeromycota fungi (Tisserant *et al*., 2013).

### Cytology of the colonization under contrasting [CO_2_]_a_

Our physiological data closely match the cytological changes that occur in plants under elevated [CO_2_]_a_ and may be interpreted in the light of increased photosynthate allocation to the fungal partners in mesh cores, either as a proportion of net fixation (*T. lacunosa*; Fig. 3a) or as a total amount of carbon allocated (*H. gibbsiae*; Fig. 3b).

The absence of thick-walled structures (interpreted as fungal spores by Carafa *et al*., 2003; Duckett *et al*., 2006; Pressel *et al*., 2010) in both liverwort genera and the necrosis of the intercellular hyphal system in *T. lacunosa* at elevated [CO_2_]_a_ suggest that the fungus is under less stress and may be allocating more easily acquired carbon into mycelial networks rather than investing in perennating structures. This idea is further corroborated by the presence of numerous, thin-walled hyphae in the mucilage that surrounds the underground axes of *H. gibbsiae* (Fig. 7g). Thus, under elevated [CO_2_]_a_ the foraging activities of the fungus in the soil increase, consistent with the substantial increase in plant photosynthate allocated to the fungus (Fig. 3b).

The large difference between *T. lacunosa* and *H. gibbsiae* in total carbon allocation to fungal partners at high [CO_2_]_a_ (Fig. 3b) may correlate with major morphological and cytological differences in the two plant hosts. *Treubia lacunosa* possesses rhizoids and therefore is likely to take up nutrients directly from the soil. As it lacks rhizoids completely, *H. gibbsiae* is likely to be highly dependent on its symbiont for phosphorus and other soil nutrients. While *T. lacunosa* grows superficially with its entire thalli being photosynthetic (Fig. 2a), over half of the total biomass of *H. gibbsiae* comprises subterranean, fungus-containing axes which also possess a cylinder of food-conducting cells around the central water-conducting cells (Ligrone *et al*., 2012). Both kinds of conducting cell are absent in *T. lacunosa*. The translocation system in *H. gibbsiae* may reflect dependence of the plant on fungal-acquired nutrients and the need to supply its fungus with carbohydrates more effectively than *T. lacunosa* (Fig. 3b).

The functional roles of the various fungal structures inside Haplomitriopsida liverworts remain to be elucidated. Arbuscules in thalloid liverworts such as *Marchantia* and *Targionia* have hyphal diameters of 1.0–1.5 and 0.5–0.8 μm, respectively (Strullu-Derrien *et al*., 2014), which are similar to those of the hyphal coils in *H. gibbsiae* and *T. lacunosa* (0.5–1.0 μm). But the latter, with their limited branching, present a much more restricted fungus–plant surface area. The cytology typical of storage organs of young lumps with subsequent accumulation of an abundant interfacial matrix and proliferation of host cytoplasmic organelles suggests that these structures may be involved in active metabolic interactions with the host (Carafa *et al*., 2003; Duckett *et al*., 2006). Previous studies have indicated that the fungal lumps in both *Treubia* and *Haplomitrium* have a shorter lifespan than the hyphal coils (Carafa *et al*., 2003; Duckett *et al*., 2006). In arbuscular mycorrhizal symbioses the life span of arbuscules depends on their ability to deliver nutrients to the host and is regulated by host plant demand (Javot *et al*., 2007; Kiers *et al*., 2011). Plants may be able to discriminate between efficient and inefficient fungi by maintaining efficient arbuscules and eliminating inefficient ones through early degradation (Parniske, 2008). Further studies to determine exactly where the bidirectional exchange of nutrients occurs (i.e. symbiotic interface) in plant–Mucoromycotina symbioses should now be a priority.

The Haplomitriopsida lineage is estimated to have diverged from the rest of the liverworts in the early Devonian (Heinrichs *et al*., 2007) over 400 Ma and is the only group of land plants known to date that appear to associate exclusively with members of the Mucoromycotina, while all other lineages that harbour Mucoromycotina fungi also form Glomeromycota associations (Bidartondo *et al*., 2011) (Fig. 1a). Morphologically, *Haplomitrium* and *Treubia* are unique amongst liverworts (Carafa *et al*., 2003; Duckett *et al*., 2006). The characteristic fungal lump structures in these genera appear to not be produced in any other early divergent land plants, extant (Bidartondo *et al*., 2011; Desiró *et al*., 2013) or extinct (Strullu-Derrien *et al*., 2014), that either associate, or are thought to associate, with members of the Mucoromycotina. The substantial anatomical changes induced in the hosts by fungi (see Figs 8) are also unique to the Haplomitriopsida–Mucoromycotina partnership. Our observations that asymbiotic thalli of *T. lacunosa* lack the system of mucilage-filled intercellular spaces completely and *H. gibbsiae* fails to develop mucilaginous leafless axes indicate that the copious production of mucilage unique to these two liverworts is induced by their fungal partners (Fig. 2c,d).

### Evolutionary considerations

The present demonstration that the earliest divergent liverwort lineage – the Haplomitriopsida – forms mutualistic partnerships with Mucoromycotina fungi now invites novel hypotheses on the evolution of land plant–fungus symbioses and their role in the conquest of Earth's land-masses. Our discovery, coupled with studies showing that a wide range of extant nonvascular land plants form symbiotic associations with both Mucoromycotina and Glomeromycota fungi, sometimes simultaneously, points to much more versatile and shifting plant–fungal evolutionary scenarios than hitherto assumed.

There remain considerable uncertainties in the timing of the origins of the key players and components involved in the establishment of land-plant fungal symbioses, with palaeontological data and molecular clock date estimates often differing greatly. However, the SYM genes controlling mycorrhiza formation in angiosperms are distributed across land plants, including bryophytes and basal liverworts (Wang *et al*., 2010). In fact, the mycorrhiza-formation genes from Haplomitriopsida recover the Glomeromycota mycorrhizal phenotype, including vesicles and arbuscles, in a transformed mutant of the angiosperm *Medicago truncatula*, despite Haplomitriopsida not hosting Glomeromycota fungi. This suggests Mucoromycotina associations in liverworts could be ancestral, or at least as ancestral as those with Glomeromycota fungi, and reveals that the genes required for symbiosis have been conserved between the liverworts that associate with Mucoromycotina, through to higher plants that associate with Glomeromycota.

The ability to engage in partnerships with both Glomeromycota and Mucoromycotina fungi, sometimes simultaneously, observed thus far in some hornwort (Desiró *et al*., 2013) and liverwort species may be an ancient strategy. For the structurally simpler ‘lower’ land plants this could be a form of ‘bet-hedging’, whereby the plant has a choice between obligate symbionts and facultative ones, or engaging with both simultaneously. There may also be other, thus far uninvestigated benefits for plant–Mucoromycotina symbiosis such as protection against biotic or abiotic stresses. Ecological, physiological and cytological studies on plants known to harbour both types of symbiont are now needed to unravel the trade-offs associated with such symbiotic flexibility.

## Supporting Information

Fig S1 Illustration of the experimental procedure.

Table S1 Artificial rainwater solution **Table S2** Fungal sequence data from experimental plants collected from the field
