# Supplementary material for: First evidence of mutualism between ancient plant lineages (Haplomitriopsida liverworts) and Mucoromycotina fungi and its response to simulated Palaeozoic changes in atmospheric CO2
Source: New Phytol. 2014 Sep 17;205(2):743–56. doi: 10.1111/nph.13024 (PMC4303992; doi:10.1111/nph.13024)
Supplement: Fig S1 — Illustration of the experimental procedure. [file nph0205-0743-sd1.docx]

**Supporting Information Fig. S1**

**First evidence of mutualisms between ancient plant lineages (Haplomitriopsida liverworts) and Mucoromycotina fungi and its response to simulated Palaeozoic changes in atmospheric CO_2_**


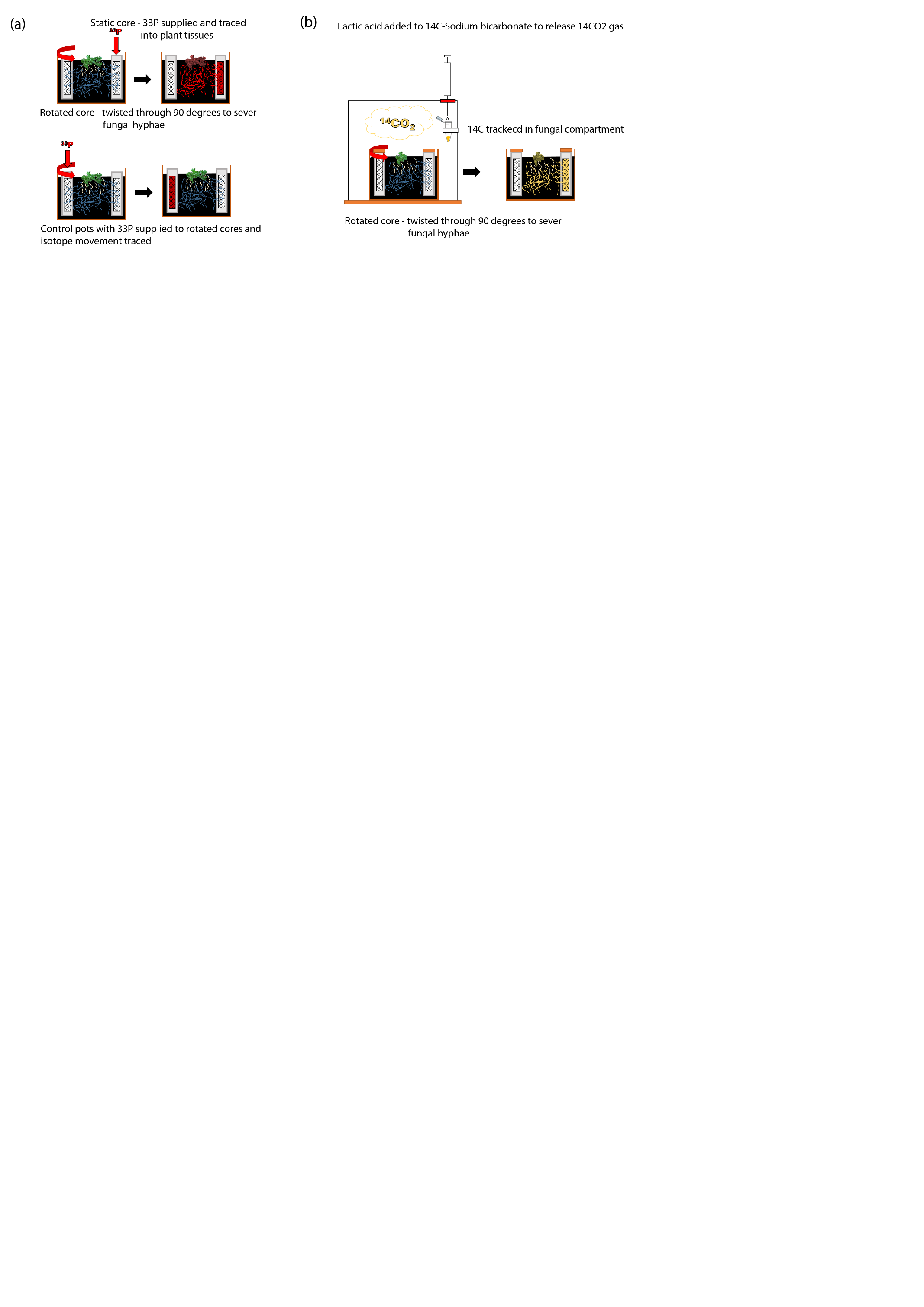


**Fig. S1** Illustration of experimental procedure for labelling with **(a)** ^33^P-orthophosphate and **(b)** ^14^CO_2._
