# Supplementary material for: First evidence of mutualism between ancient plant lineages (Haplomitriopsida liverworts) and Mucoromycotina fungi and its response to simulated Palaeozoic changes in atmospheric CO2
Source: New Phytol. 2014 Sep 17;205(2):743–56. doi: 10.1111/nph.13024 (PMC4303992; doi:10.1111/nph.13024)
Supplement: Table S1 — Artificial rainwater solution Table S2 Fungal sequence data from experimental plants collected from the field [file nph0205-0743-sd2.docx]

**Supporting Information Table S1 & S2**

**First evidence of mutualisms between ancient plant lineages (Haplomitriopsida liverworts) and Mucoromycotina fungi and its response to simulated Palaeozoic changes in atmospheric CO_2_**

**Table S1** Artificial rainwater (Olsson &Tyler, 1993) solution. Rainwater solution was applied to experimental systems as a fine mist every two days. Plants were misted until the surface of the plant was visibly wet.

|  | **mg l^-1^** | **In 10 l (g)** |
| --- | --- | --- |
| NH_4_NO_3_ | 5.6 | 0.058 |
| NaCl | 4.3 | 0.043 |
| MgSO_4_.7H_­2­_O | 2.1 | 0.023 |
| H_2_SO_4_ (µl l^-1^) | 30 | 300 µl |
| NaH_2_PO_4_.2H_2_O | 0.47 | 0.0047 |
| K_2_SO_4_ | 1.2 | 0.012 |
| CaCl | 4.2 | 0.042 |

**Table S2** Fungal sequence data

| Fungal sequence from field-collected plants used in experiments | TTTATACTGGTGAAACTGCGAATGGCTCATTAAATCAGTTATCGTTTATTTGATAATACCTTACTACTTGGATAACCGTGGTAATTCTAGAGCTAATACATGCTAAAAATCCCGACTTCTGGAAGGGATGTATTTATTAGATAAAAAACCAATGCGGGCAACCGCTTCTCTGGTGATTCATAATAACTTTTCGAATCGTATGGCCTTGCGCTGACGATGATTCATTCAAATTTCTGCCCTATCGACTTTCGATGGTAGGATAGAGGCCTACCATGGTTTTTACGGGTAACGGGGAATTAGGGTTCGATTCCGGAGAGGGAGCCTGAGAAACGGCTACCACATCCAAGGAAGGCAGCAGGCGCGCAAATTACCCAATCCCGATACGGGGGGGTAGTGACAATAAATAACAATACAGGGCCCTTTCGGGTCTTGTAATTGGAATGAGTACAATTTAAATCCCTTAACGAGGAACAATTGGAGGGCAAGTCTGGTGCCAGCAGCCGCGGTAATTCCAGCCCCAATAGCGTATATTAAAGTTGTTGCAGTTAAAAAGCTCGTAGTTGAATTTTAGCCTTGGCTGGGCGGTCCGGCCTCACGGTCGGTACTGCTTTGGTTGGGGTTCACCTTCTGGTGAGCCAGCATGCTCTTAACTGGGTGTGTTGGGGAACCAGGACTTTTACTTTGAAAAAATTAGAGTGTTTAAAGCAGGCATCCGCTTGAATACATTAGCATGGAATAATGGAATAGGACCTTGGTTCTATTTTGTTGGTTTCTAGGACCATAGTAATGATTAATAGGGATAGTTGGGGGTATTAGTATTTAATTGTCAGAGGTGAAATTCTTGGATTTATGAAAGACTAACTTCTGCGAAAGCATTTACCAAGGATGTTTTCATTAATCAAGAACGAAAGTTAGGGGATCGAAGACGATCAGATACCGTCGTAGTCTTAACCATAAACTATGCCGACTAGGGATCGGACGATGTTATTTTTTGTGACTCGTTCGGCACCTTGAGAGAAATCAAAGTTTTTGGGTTCCGGGGGGAGTATGGTCGCAAGGCTGAAACTTAAAGGAATTGACGGAAGGGCACCACCAGGAGTGGAGCCTGCGGCTTAATTTGACTCAACACGGGGAAACTCACCAGGTCCAGACATAGTAAGGATTGACAGATTGAGAGCTCTTTCTTGATTCTATGGGTGGTGGTGCATGGCCGTTCTTAGTTGGTGGAGTGATTTGTCTGGTTAATTCCGTTAACGGACGAGACCTTAACCTGCTAAATAGTTAGGCCAACTTTTAGTTGGTCGCCAACTTCTTAGAGGGACTATTGACGTTTAGTCAATGGAAGTTTGAGGCAATAACAGGTCTGTGATGCCCTTAGATGTTCTGGGCCGCACGCGCGCTACACTGACGAAGTCAGCAAGTCTATAACCTTGGCCGGAAGGTCTGGGTAATCTTTTGAAACTTCGTCGTGCTGGGGATAGAGCATTGCAATTATTGCTCTTCAACGAGGAATTCCTAGTAAGCGTGAGTCATCAGCTCGCGTTGATTACGTCCCTGCCCTTTGTACACACCGCCCGTCGCTACTACCGATTGAATGGCTTAGTGAGTCCCTCGGATTGAAGCCCAGAAGCTGGCAGCAGCATCCGGGTGTTGAGAAGTTGGGCAAACTTGGTCATTTTAGAGGA |
| --- | --- |

**References**

**Olsson PA, Tyler G.** **1993.** Occurrence of non‐mycorrhizal plant species in south Swedish rocky habitats is related to exchangeable soil phosphate. *Journal of Ecology* **92:** 808–815.
